# Supplementary material for: On critical dynamics and thermodynamic efficiency of urban transformations
Source: R Soc Open Sci. 2018 Oct 17;5(10):180863. doi: 10.1098/rsos.180863 (PMC6227941; doi:10.1098/rsos.180863)
Supplement: Supplemental text [file rsos180863supp1.pdf]

On critical dynamics and thermodynamic efficiency of urban transformations

Supplemental Material

Emanuele Crosato[1,2,*], Ramil Nigmatullin[1], and Mikhail Prokopenko[1]

[1]Complex Systems Research Group and Centre for Complex Systems, Faculty of Engineering and IT, The University of Sydney, Sydney, NSW 2006, Australia.
[2]CSIRO Data61, PO Box 76, Epping, NSW 1710, Australia.
[*]emanuele.crosato@sydney.edu.au

1 Greater Sydney and data sources

Greater Sydney is an urban area covering more that 12,000 square kilometres, delimited in all directions by either the Pacific Ocean or by the several surrounding national parks. It includes the City of Sydney as well as other urban agglomerations such as Parramatta, Penrith, Campbelltown and Gosford, for a total population of approximately 5 million (see Fig. S1(a)). According to 2011 Census data, the working population of Greater Sydney is 1.8 million. People daily commute between residence areas (or suburbs), where they live, and employment areas, where they work. The territory is partitioned into 270 residence areas and 2,156 employment areas. The data used for this study was provided by the Australian Bureau of Statistics. This includes the geospatial data of the areas of employment and residence, as well as the Census data for year 2011.

The employment areas are defined by the standard Destination Zone (DZN), which was designed by the New South Wales transport authority in order to spatially classify employment places, with the purpose of analysing commuting data and developing transport policies. The standard Statistical Area Level 2 (SA2), as defined by the Australian Statistical Geography Standard, was used for the residence areas. Geographical areas of level SA2 represent small communities that closely interact socially and economically. The population of these areas can vary from 3,000 to 25,000 individuals, with an average population of 10,000 individuals. A satellite map showing the DZN and SA2 areas partitioning Greater Sydney is provided Fig. S1(b-d).

The Census data for year 2011 was geographically classified by the Australian Bureau of Statistics in accordance with the both geographical areas DZN and SA2, and included the travel-to-work matrix T_{ij}, the average weekly income I_i and the average weekly rent R_j, for all DZN areas i and SA2 areas j. The Census data also included the amount of people who work in food retailing stores (including supermarkets, grocery stores, meat and fish stores, fruit and vegetables stores and liquor stores) that are located in specific SA2 areas. This data was utilised to estimate the amount of goods, matching the services S_j available in each residence area.

The cost of travelling C_{ij} was estimated as the Euclidean distance between the centres of the employment and residence areas. An alternative approach would be to calculate the time of travelling using, for example, Google Maps or OpenStreetMap data. However, in this case one needs to assemble a dataset with several Sydney transport networks (bus, rail and ferry), in addition to the road networks. While the latter data are publicly available (e.g., OpenStreetMaps), the former is not. Furthermore, the network connectivity needs to be augmented with price of the corresponding travels, as well as individual preferences reflected in travel pattern data (such as Opal cards). A simpler solution based on Euclidean distances appears to be less biased than a more sophisticated but incomplete approach.

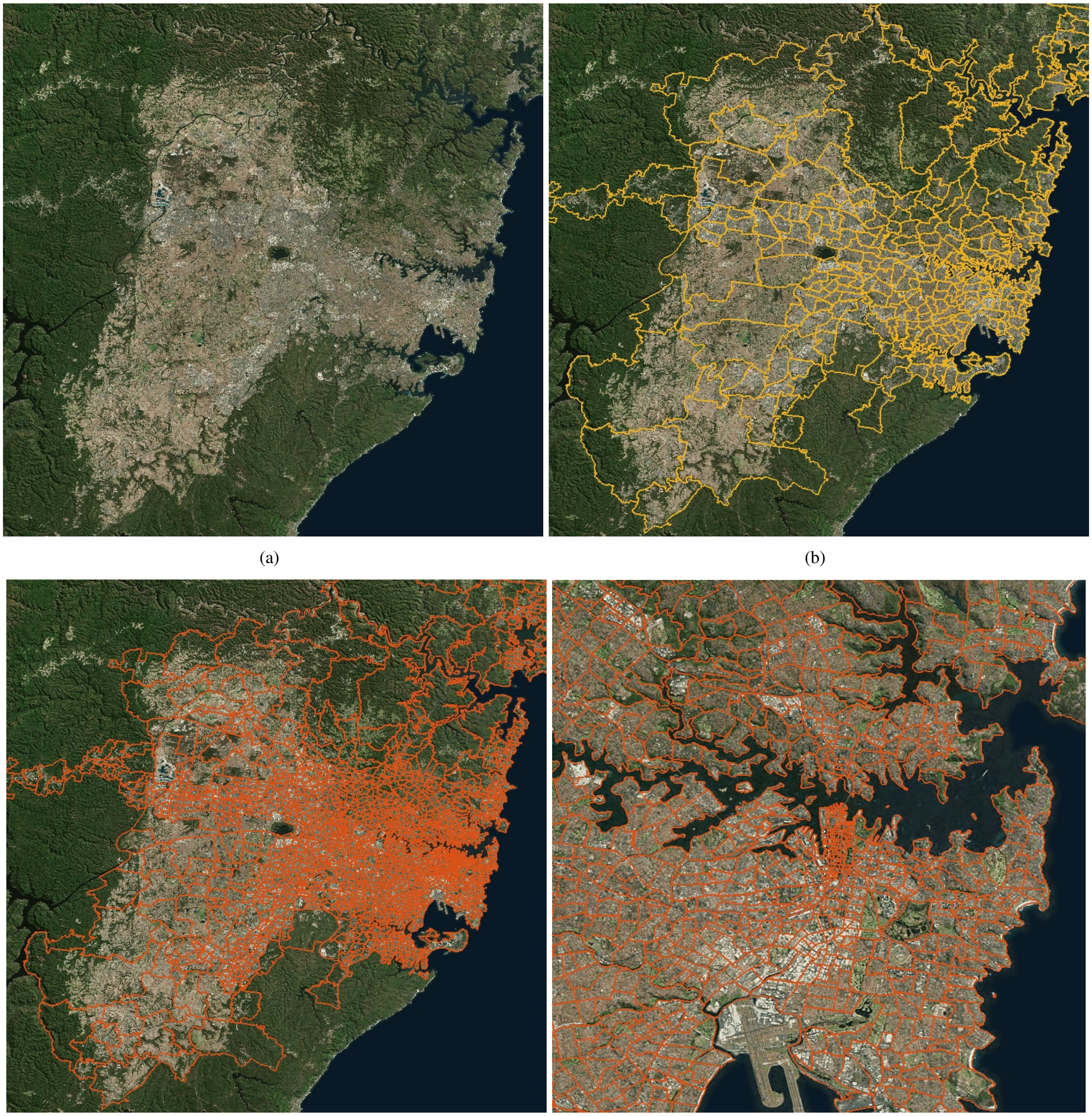

(a) (b) (c) (d)

Figure S1: (a) Satellite image of Greater Sydney (TerraColor imagery by Earthstar Geographics LLC). The satellite image showing the areas of residence (b), according with the standard SA2, and the areas of employment (c), according with the standard DZN. (d) A magnification showing the employment areas of the City of Sydney, including City Business District (CBD).

In summary, the constraints used in Eq. (2.2) and Eq. (2.3) of the Boltzmann component are produced from the Census data, given the definition of attractiveness $A_j = \log(f(P_j)\, S_j)$ in terms of the population P_j and the services S_j, while the constraint used in Eq. (2.4) is produced by the geospatial data.

2 Calibration of the model

The model was calibrated by identifying the optimal values $\hat{\alpha}$ and $\hat{\gamma}$ for which the output $\mathcal{Y}^0_{ij}$ best matches the actual flow of income Y_{ij} of Sydney-2011. The difference between actual and predicted flow of income was estimated as the sum of (the absolute values of) the differences between all values of the matrices $\mathcal{Y}^0_{ij}$ and Y_{ij}, which is $e(\mathcal{Y}^0_{ij}) = \sum_i \sum_j \left|\mathcal{Y}^0_{ij} - Yij\right|$. The result is shown in Fig. S2.

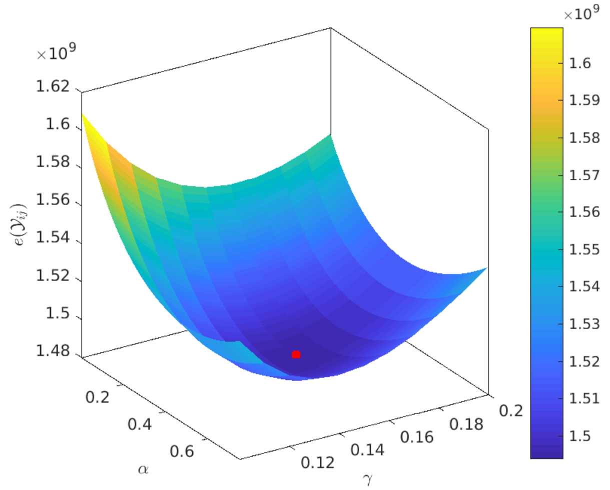

Figure S2: Calibration of the parameters α and γ. The horizontal axes represent values of α and γ within the considered ranges, while the vertical axis represents the difference between the income flow $\mathcal{Y}^0_{ij}$ produced by the model and the actual income flow Y_{ij} given by Sydney-2011 Census data, calculated as $e(\mathcal{Y}^0_{ij}) = \sum_i \sum_j \left|\mathcal{Y}^0_{ij} - Yij\right|$. The red dot represents the optimal values $\hat{\alpha}$ and $\hat{\gamma}$.

3 Thermodynamic analysis

Let us consider the state functions $X_m(x)$ that describe a physical system over its configurations x. In a stationary state, the Gibbs measure defines the probability of the states of the system:

$$p(x|\theta) = \frac{1}{Z(\theta)} e^{-\beta H(x,\theta)} = \frac{1}{Z(\theta)} e^{-\sum_m \theta_m X_m(x)}, \tag{1}$$

where θ_m are thermodynamic variables, $\beta = 1/k_b T$ is the inverse temperature T (k_b is the Boltzmann constant), $H(x,\theta)$ is the Hamiltonian defining the total energy at state x, and $Z(\theta)$ is the partition function [1, 2]. The Gibbs free energy of such system is:

$$G(T, \theta_m) = U(S, \phi_m) - TS - \phi_m \theta_m, \tag{2}$$

where U is the internal energy of the system, S is the configuration entropy and ϕ_m is an order parameter. Let us also consider the generalised internal energy U_{gen} in the sense of Jaynes [3], such that

$$\langle \beta U_{gen} \rangle = U(S, \phi_m) - \phi_m \theta_m, \tag{3}$$

where the angle brackets represent average values over the ensemble. The generalised first law holds $\langle \beta U_{gen} \rangle = \langle \beta Q_{gen} \rangle + \langle \beta W_{gen} \rangle$, where Q_{gen} and W_{gen} are, respectively, the generalised heat and the generalised work.

The Fisher information [4] measures the amount of information that an observable random variable X carries about an unknown parameters $\theta = [\theta_1, \theta_2, \ldots, \theta_M]^T$. If $p(x|\theta)$ is the probability of the realisation x of X given the parameters θ, the Fisher information matrix is defined as

$$F_{mn}(\theta) = E\left[\left(\frac{\partial \ln p(x|\theta)}{\partial \theta_m} \right) \left(\frac{\partial \ln p(x|\theta)}{\partial \theta_n} \right) \middle| \theta \right], \tag{4}$$

where the function $E(y)$ is the expected value of y. For a physical system described by the Gibbs measure in (1), the Fisher information has several physical interpretations, e.g., it is equivalent to the thermodynamic metric tensor $g_{mn}(\theta)$, is proportional to the second derivative of the free entropy $\psi = \ln Z = -\beta G$, and to the derivatives of the corresponding order parameters with respect to the collective variables [1, 5, 6, 2, 7]:

$$F_{mn}(\theta) = g_{mn}(\theta) = \frac{\partial^2 \psi}{\partial\theta_m \partial\theta_n} = \beta \frac{\partial \phi_m}{\partial \theta_n}. \quad (5)$$

Furthermore [8],

$$F(\theta) = \frac{d^2 S}{d\theta^2} - \frac{d^2 \langle \beta U_{gen} \rangle}{d\theta^2}. \quad (6)$$

Under a quasi-static protocol the total entropy production is zero, and therefore any change in the configuration entropy due to the driving process is matched by the flow of heat to the environment:

$$\frac{dS}{d\theta} = \frac{d\langle \beta Q_{gen} \rangle}{d\theta}. \quad (7)$$

Thus, combining (6) and (7) with the first law of thermodynamics yields another important result for the generalised work W_{gen} [8]:

$$F(\theta) = -\frac{d^2 \langle \beta W_{gen} \rangle}{d\theta^2}. \quad (8)$$

4 Entropy and a proxy of order parameter

A higher entropy indicates a more homogeneous distribution of the income to all suburbs, while a lower entropy indicates a less balanced distribution of the income biased towards one or few suburbs. We observe that the entropy decreases with both parameters α and γ (see Fig. S3). This behaviour is expected and has a clear interpretation. If the social disposition α is low, people have modest preference for attractive suburbs and thus settle (and move their income) more homogeneously within the region, while if α is high people tend to aggregate around the areas with the higher attractiveness. Similarly, if travel impedance γ is low people are less concerned about high travel costs, and therefore can settle at any distance from their work place, while if γ is high people prefer to live closer to their employment areas to incur lower commuting costs.

To formalise this intuition, one typically introduces and traces corresponding order parameters. This is however hindered by an incomplete statistical-mechanical description of the system, and we first illustrate a simpler approach which considers a proxy of an order parameter. Such a proxy characterises the equilibrium distribution of the services S_j, for different

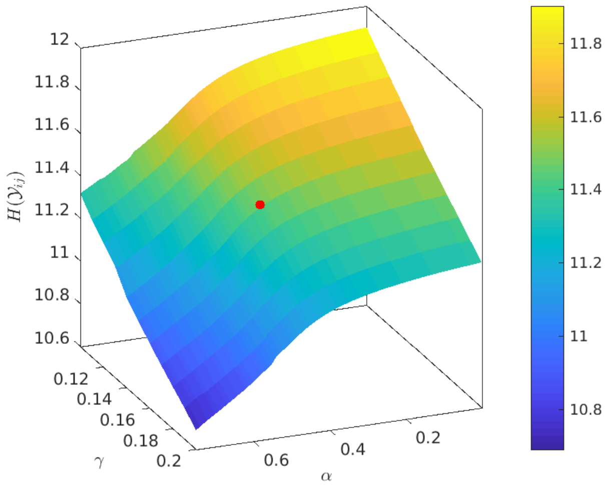

Figure S3: Entropy of $\mathcal{Y}^*_{ij}$ after the services S_j have evolved to reach an equilibrium. The horizontal axes represent values of α and γ within the considered ranges, while the vertical axis represents the entropy $H(\mathcal{Y}_{ij})$ at corresponding values of α and γ. The red dot indicates the combination of $\hat{\alpha}$ and $\hat{\gamma}$ for which $\mathcal{Y}^0_{ij}$ best matches Sydney-2011 Census data.

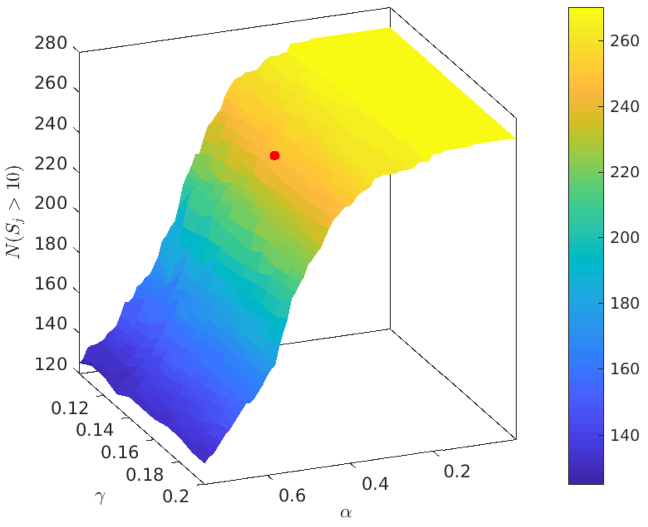

Figure S4: The number of suburbs $N(S_j > 10)$ with more than 10 units of services available, after the services S_j have evolved to reach an equilibrium. The horizontal axes represent values of α and γ within the considered ranges, while the vertical axis represents $N(S_j > 10)$ at corresponding values of α and γ. The red dot indicates the combination of $\hat{\alpha}$ and $\hat{\gamma}$ for which $\mathcal{Y}_{ij}^0$ best matches Sydney-2011 Census data.

values of α and γ, in terms of the number of suburbs in which the amount of available services exceeds a threshold, i.e., "services-abundant" suburbs. Fig. S4 shows the number of services-abundant suburbs, that is $N(S_j > 10)$, for different values of α and γ, after the urban evolution has converged. Again, as with the entropy dynamics, the variation of γ does not greatly affect the number of services-abundant suburbs. On the contrary, this number displays an abrupt change with respect to α: for low values of the social disposition all 270 suburbs are services-abundant, but as α increases the number of services-abundant suburbs reduces quickly past a specific value of α. At high values of social disposition approximately 120 residence areas remain services-abundant.

Fig. S3 and Fig. S4 also show the values $\hat{\alpha}$ and $\hat{\gamma}$ which best matches Sydney-2011 Census data (the red dot on either the entropy or the $N(S_j > 10)$ surfaces). This value is within a close proximity to the social disposition where the abrupt change is observed.

References

[1] Brody DC, Rivier N. Geometrical aspects of statistical mechanics. Physical Review E. 1995;51:1006–1011.

[2] Crooks GE. Measuring Thermodynamic Length. Physical Review Letters. 2007;99:100602.

[3] Jaynes ET. Information Theory and Statistical Mechanics. Physical Review. 1957;106:620–630.

[4] Fisher RA. On the Mathematical Foundations of Theoretical Statistics. Philosophical Transactions of the Royal Society of London A: Mathematical, Physical and Engineering Sciences. 1922;222(594-604):309–368.

[5] Brody DC, Ritz A. Information geometry of finite Ising models. Journal of Geometry and Physics. 2003;47(2):207–220.

[6] Janke W, Johnston DA, Kenna R. Information geometry and phase transitions. Physica A: Statistical Mechanics and its Applications. 2004;336(1–2):181–186.

[7] Prokopenko M, Lizier JT, Obst O, Wang XR. Relating Fisher information to order parameters. Physical Review E. 2011;84:041116.

[8] Crosato E, Spinney RE, Nigmatullin R, Lizier JT, Prokopenko M. Thermodynamics and computation during collective motion near criticality. Physical Review E. 2018;97:012120.
